# Vestibular perception, balance impairment, and fall risk in community-dwelling older adults

**DOI:** 10.1093/ageing/afag216

**Published:** 2026-07-29

**Authors:** Yuxiao Li, Zaeem Hadi, Rebecca M Smith, Barry M Seemungal, Toby Jack Ellmers

**Affiliations:** Department of Brain Sciences, Centre for Vestibular Neurology, Imperial College London, London, W6 8RF, UK; Department of Brain Sciences, Centre for Vestibular Neurology, Imperial College London, London, W6 8RF, UK; Department of Brain Sciences, Centre for Vestibular Neurology, Imperial College London, London, W6 8RF, UK; Department of Brain Sciences, Centre for Vestibular Neurology, Imperial College London, London, W6 8RF, UK; Department of Brain Sciences, Centre for Vestibular Neurology, Imperial College London, London, W6 8RF, UK

**Keywords:** vestibular agnosia, vestibular perceptual thresholds, vestibulo-ocular reflex, postural sway, fall risk, older people

## Abstract

**Background:**

Vestibular complaints are common in older adults and are linked to imbalance and falls. Some older adults show impaired vestibular perception despite preserved peripheral-reflex [‘vestibular agnosia (VA)’]. Yet it remains unclear if VA is independently linked to imbalance and falls in otherwise healthy older adults. We therefore investigated the prevalence of VA in community-dwelling older adults, and examined its association to balance and prospective falls.

**Methods:**

Vestibular perceptual thresholds were measured during yaw-plane rotational chair testing. Postural sway and instrumented Timed-Up-and-Go were assessed using wearable sensors, and falls were recorded prospectively over six-months. VA was identified using *K*-means clustering. Multivariable regressions examined associations between perceptual thresholds and balance outcomes; logistic and negative binomial regressions evaluated associations with prospective falls.

**Results:**

Among 166 participants (75.4 years; 81.9% female), 18.7% were classified as having VA. These individuals had worse cognition and somatosensation, and higher anxiety. Elevated (i.e. worse) vestibular perceptual thresholds were independently associated with greater sway velocity when standing on foam with eyes-open (adjusted *β* = 0.002, *P* = .03). Associations with other balance outcomes were attenuated after adjustment. Vestibular perceptual thresholds were not associated with prospective falls (odds of ≥1 fall: adjusted OR = 0.99, *P* = .65; fall counts: adjusted IRR = 1.02, *P* = .35).

**Conclusions:**

Approximately one-fifth of healthy older adults exhibit VA. While elevated perceptual thresholds are independently associated with poorer balance, they did not predict falls. Further research is needed to determine whether vestibular perceptual testing provides additional information beyond standard balance assessments for fall-risk prediction in community-dwelling healthy older adults.

## Key Points

- Approximately one-fifth of healthy older adults had impaired vestibular perception despite intact peripheral function [i.e. ‘vestibular agnosia (VA)’].
- Older adults with VA have poorer cognition, reduced lower limb somatosensation and higher anxiety.
- Higher (i.e. worse) vestibular perceptual thresholds were independently associated with greater sway velocity on foam (eyes-open).
- Higher vestibular perceptual thresholds were associated with slower timed up-and-go and greater eyes-closed foam sway in unadjusted (but not adjusted) models.
- Vestibular perceptual thresholds did not predict prospective falls over 6 months.

## Introduction

Approximately 30% of community-dwelling older adults fall annually [1, 2], and age-related balance decline plays a central role [3]. Postural control integrates visual, proprioceptive and vestibular inputs [4], with vestibular function supporting self-motion perception and spatial orientation [5]. Vestibular perceptual thresholds to self-motion, which quantify the smallest self-motion stimulus that can be reliably perceived on a given axis [6, 7], tend to increase with age [8] and relate to poorer balance performance [9–13]. Recent work has also linked impaired vestibular perception to increased prospective falls in older adults with Type-2 diabetes [11]. However, evidence for age-related differences in vestibular perception is mixed [14], and later-life comorbidities may contribute. This highlights the importance of examining vestibular function, and its relationship with balance and falls in generally healthy community-dwelling older adults.

Impaired vestibular sensation can occur due to peripheral [15] and/or central vestibular dysfunction [16, 17]. The latter, whereby impaired vestibular sensation occurs despite vestibulo-ocular activation (indicating preserved inner ear and reflex function), is known as ‘vestibular agnosia (VA)’ [17]. While VA is well-characterised in neurological conditions (e.g. traumatic brain injury [16–18] and Parkinson’s Disease [19]), age-related VA in otherwise healthy older adults is relatively less examined. In a small study (*n* = 20), Chiarovano *et al*. [20] reported that older adults with greater postural instability fail to perceive self-motion during vestibular caloric stimulation. This suggests that a VA-like impairment in vestibular sensation may be both common and clinically relevant in later life.

Most previous studies exploring vestibular perception in older adults did not assess peripheral vestibular function, making it difficult to determine whether perceptual deficits arise from central processing or peripheral vestibular dysfunction [21]. Furthermore, the prevalence and risk factors for VA in community-dwelling older adults remain unknown. Existing research has also largely focused on associations between vestibular perception and static postural sway [9, 10], while overlooking more ecologically valid tasks like walking and turning. This is important because most falls in older adults occur during dynamic activities, whereas quiet standing accounts for only ~10% of falls [22]. Turning involves yaw-plane reorientation and continuous monitoring of head direction, processes that typically rely (at least in part) on vestibular information [23]—and perhaps also on vestibular perception. Real-world turning characteristics have also been associated with recurrent falls [24], further highlighting their clinical importance.

To address these gaps, we aimed to investigate the prevalence and associated characteristics of VA in community-dwelling older adults; and to examine whether impaired vestibular perception relates to both balance performance (static postural sway and instrumented timed up-and-go (TUG), including turning characteristics) and 6-month prospective falls, after controlling for key covariates including peripheral vestibular function. We hypothesised that VA reflects an age-related central vestibular processing impairment that disrupts the conscious self-motion perception and the utilisation of vestibular signals. We therefore predicted that VA will be associated with poorer balance (particularly in vestibular-dependent balance tasks, e.g. eyes-closed on foam and turning), and with increased prospective fall risk.

## Methods

### Participants and recruitment

We recruited community-dwelling older adults aged ≥60 years in London (April—December 2024). Exclusion criteria were: a clinical diagnosis of a progressive neurological disorder (e.g. Alzheimer’s, Parkinson’s, multiple sclerosis); the inability to walk 10 metres unaided; severe visual or auditory impairment (i.e. legally blind/deaf); and major cognitive impairment (Montreal Cognitive Assessment [MoCA] score of ≤18 [25]), to ensure participants could understand and complete the threshold tests. In addition, 20 healthy younger adults (aged 22–32 years) without neurological/balance disorders were recruited to enable comparison with normative values for the vestibulo-ocular reflex (VOR) and perceptual thresholds (see Supplementary Methods).

All participants provided written informed consent, and the study was approved by the East of England—Essex Research Ethics Committee (IRAS ID: 324021).

### Measures

#### Vestibular perceptual and vestibulo-ocular reflex thresholds

**Figure 1:**
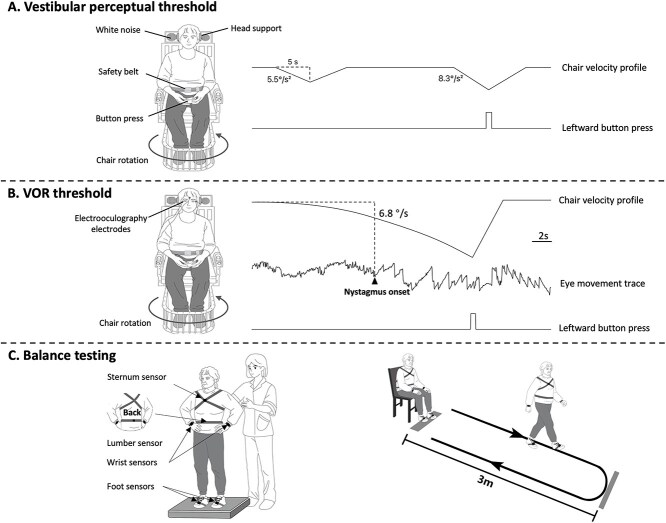
Yaw-axis rotational chair set-up and stimulus profiles for vestibular perceptual and VOR threshold measurements. A: Vestibular perceptual threshold paradigm. The upper trace shows the chair velocity profile during a constant-acceleration rotation (up to 5 s), with acceleration level adjusted across trials by the MOBS staircase algorithm (illustrated here with 5.5°/s^2^ and 8.3°/s^2^). The lower trace indicates the timing of a leftward button press; correct directional responses within the 5-s acceleration phase were scored as correct, whereas incorrect or late responses (i.e. during or after the deceleration phase) were scored as misses. B: VOR threshold paradigm. Yaw-plane rotations with linearly increasing acceleration (0.3°/s^2^ every 3 seconds). The upper trace shows the chair velocity profile, the middle trace the horizontal eye-movement response recorded with electrooculography, with the arrow indicating the onset of nystagmus, the lower trace indicates the timing of the participant’s button press to report perceived direction of rotation. C: Balance testing. Participants completed instrumented balance assessments using six inertial wearable sensors (wrists, sternum, lower lumbar and feet). For postural sway on foam: Participants stood quietly on a soft rectangular foam (50 × 41 × 6 cm, Airex®), with standardised hip-width stance and with their arms placed on their hips. For TUG, participants were asked to stand up from a chair, walk 3 metres, turn around, return and then seat back down.

Figure 1 illustrates the apparatus used to objectively assess vestibular perceptual and VOR thresholds during passive yaw-plane rotations using a vibration-free motorised rotating chair (Contraves, USA) [17, 26]. Participants were seated upright with a head support and a safety harness, with visual and auditory cues minimised (lights off, blackout curtain, white noise), and were asked to press a button (left or right) as soon as they perceived a rotation in the respective direction.

During perceptual testing (Figure 1A), the stimuli followed a constant acceleration profile, with acceleration determined by the modified binary search (MOBS) staircase algorithm [27] with independent randomly left−/right-ward rotations (≤5 s; 0.2 Hz; matched deceleration). VOR thresholds were obtained simultaneously during perceptual testing; however, because perceptual thresholds were much higher than VOR thresholds, the algorithm rapidly moved away from the peri-VOR threshold range. Therefore, we additionally assessed VOR thresholds using the same yaw-plane chair rotations with linearly increasing acceleration (0.3°/s^2^ every 3 s) [17] (Figure 1B). More detailed information regarding the chair and algorithm can be found in the Supplementary Methods.

To control for potential confounding by reaction speed and vigilance on perceptual thresholds, participants completed a computerised hand reaction time task [17].

### Balance assessments

Balance was assessed using six inertial wearable sensors (Opal™, APDM, Inc., Portland, OR, USA) (Figure 1C). For s*tatic balance*, the analyses focused on soft-surface foam conditions (50 × 41 × 6 cm, Airex® foam; eyes-open and eyes-closed for 30 seconds each [28]), as previous studies in traumatic brain injury distinguished VA groups on foam surface but not on solid surface [17]. Sway velocity was calculated as our primary outcome, with sway area calculated as a secondary outcome. Also, participants performed an *instrumented TUG test* as a measure of functional balance and mobility [29]. We derived the total duration, turn angle and turn velocity, focusing our primary TUG analyses on yaw-plane angular velocity components (turn velocity). All parameters were derived using validated algorithms within the Mobility Lab software platform [30, 31].

### Prospective falls

Participants were provided with a falls diary and instructed to list any falls experienced over 6-month follow-up, with entries verified by telephone and/or email at 3 and 6 months. Falls were assessed as all-cause events using the World Health Organization definition: ‘an event which results in a person coming to rest inadvertently on the ground, floor or other lower level’ [32]. Fall occurrence was recorded as a yes/no outcome.

### Demographics and health-related variables

Age, sex, ethnicity, education, hypertension and diabetes, number of medications, smoking, alcohol use, previous falls and dizziness history in last 12-month were collected. Validated questionnaires were also completed as baseline: Short Falls Efficacy Scale–International (FES-I) [33]; Hospital Anxiety and Depression Scale (HADS) [34], MoCA [25], Trail Making Test Part B (TMT-B) [35], Dizziness Handicap Inventory [36] and Visual Vertigo Analogue Scale [37]. Anthropometric data included body mass index (BMI), lower limb strength (5-times sit-to-stand test) [38], ankle vibration thresholds [39, 40]. More details are provided in the Supplementary Methods.

### Statistical analysis

Between-group comparisons used independent *t*-tests or Mann–Whitney U tests (continuous) and χ^2^ tests (categorical). Baseline *P*-values were unadjusted and exploratory. All statistical tests were two-sided, with significance set at *P <* .05. Analyses were conducted using SPSS (version 29.0) and R software (Version 4.4.2).

We used complete-case analyses, excluding participants with missing outcomes from the aim-specific analyses. For ***Aim 1***, because VA lacks a formal consensus diagnostic criteria, we used a two-step operational classification approach to identify a VA-like phenotype. Since VA is characterised by impaired perceptual sensitivity in the context of intact peripheral vestibular function, we first excluded participants with evidence of both elevated perceptual and VOR thresholds to avoid misclassifying possible peripheral sensory loss as VA [21] (see the Supplementary Methods for further details on this exclusion process). These participants were categorised descriptively as the ‘concurrent perceptual–VOR elevation’ group. Among the remaining participants, *K*-means clustering (as previously used to classify VA [16, 18]) was then applied as a data-driven subgrouping tool based on age and averaged left/right perceptual thresholds (but see Supplementary Figure 1 for identical clusters when instead based on left and right perceptual thresholds), in order to quantify the prevalence of VA and conduct between-group comparisons of demographic data. Aims 2 and 3 did not rely on this classification; instead perceptual thresholds were modelled continuously.

For ***Aim 2***, we used multivariable linear regression to test associations between perceptual thresholds and balance outcomes, reporting unadjusted and fully adjusted models [age, sex, MoCA, VOR thresholds, ankle vibration thresholds and 5-times sit-to-stand (indication of lower limb strength)]. Please see Supplementary Table 4 for additional sensitivity analyses with the removal of 5-times sit-to-stand as a covariate (given the overlap between this and components of TUG).

***Aim 3*** analysed whether vestibular perceptual thresholds predicted 6-month prospective falls using logistic regression (≥1 fall; yes/no) and negative binomial regression (fall counts). We fitted unadjusted and covariate-adjusted models (VOR thresholds, age, sex, prior falls, ankle vibration thresholds and 5-times sit-to-stand duration). Sample size was estimated for the planned multivariable logistic regression analysis of prospective falls. Assuming an OR of 2.0 for the association between vestibular perceptual thresholds and falls (a conservative estimation based on [11]), an expected falls prevalence of approximately one-third, and seven predictors in the model. This required ~160 participants with follow-up data.

Detailed descriptions of the analyses for each aim (and additional sensitivity analyses) are provided in the Supplementary Methods.

## Results

### Participant characteristics

**Figure 2:**
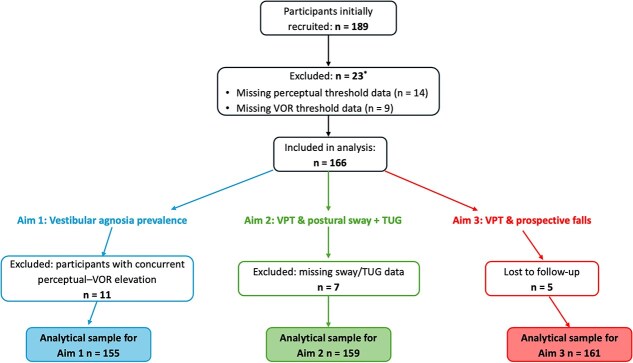
Flow of participants through research aims. ^*^Missing perceptual threshold data was most commonly due to dizziness associated with the testing procedure (i.e. dizziness either evoked by the rotations themselves, or participants’ recent dizziness meaning that they did not wish to risk re-eliciting symptoms—Noting that all participants with recent dizziness (past 2 weeks) were tested with Dix-Hallpike manoeuvre to rule out current BPPV). Missing VOR threshold data due to poor recording quality or participants did not complete the testing. Abbreviations: VOR, vestibulo-ocular reflex; VPT, vestibular perceptual threshold; TUG, timed-up-go test.

Of the 189 participants recruited, 14 were excluded for missing vestibular perceptual threshold data (our primary variable) and additionally 9 for missing VOR threshold (see flow-diagram, Figure 2), leaving 166 participants included (Table 1). The mean age was 75.4 ± 6.2 years (range 62–90), 81.9% were female, and 43.3% reported falling in the past year. The median vestibular perceptual and VOR thresholds were 8.6°/s (IQR: 4.95–12.73) and 4.14°/s (IQR: 2.46–6.46), respectively.

**Table 1:** Baseline characteristics of participants.

Variable	All ^a^ (*n* = 166)	VA- group (*n* = 126; 81.3%)	VA+ group (*n* = 29; 18.7%)	*P*
**Demographics**				
Age (years), mean ± SD	75.4 ± 6.2	75.0 ± 6.3	76.5 ± 6.2	.23
Female sex, n (%)	136 (81.9%)	104 (82.5%)	25 (86.2%)	.84
BMI (kg/m^2^), mean ± SD	25.55 ± 4.15	25.72 ± 4.05	25.38 ± 4.79	.69
Ethnicity: White, n (%)	152 (91.6%)	116 (92.1%)	25 (86.2%)	.53
Educational level, n (%)				.37
Non-tertiary	77 (46.4%)	58 (46.0%)	16 (55.2%)	–
University or higher	89 (53.6%)	68 (54.0%)	13 (44.8%)	–
**Sensory and Vestibular Function**				
Perceptual threshold (°/s), median (IQR)	8.60 (4.95, 12.73)	6.28 (4.45, 9.70)	20.40 (16.13, 24.45)	**<.001**
VOR threshold (°/s), median (IQR)	4.14 (2.46, 6.46)	3.31 (2.24, 5.55)	5.40 (3.76, 7.08)	**.006**
Hand reaction time (s), median (IQR)	0.51 (0.45, 0.59)	0.52 (0.45, 0.57)	0.52 (0.45, 0.63)	.89
Ankle vibration threshold, median (IQR)	6.5 (4.7, 8.0)	6.6 (5, 8)	6.0 (3.8, 7.5)	**.02**
**Medical History**				
Hypertension, n (%)	51 (30.7%)	40 (31.7%)	10 (34.5%)	.78
Diabetes, n (%)	5 (3.0%)	4 (3.2%)	1 (3.4%)	.94
No. medications ≥4, n (%)	37 (22.3%)	26 (20.6%)	9 (31.0%)	.23
Smoking history (smoke or quitted), n (%)	80 (48.2%)	61 (48.4%)	15 (51.7%)	.75
Alcohol use, n (%)	117 (70.5%)	92 (73.0%)	18 (62.1%)	.24
**Fall history**				
Past 12-month fall history, n (%)	71 (43.3%)	50 (40.0%)	13 (46.4%)	.53
Short FES-I, median (IQR)	9 (8, 11)	9 (7, 11)	10 (8, 12)	.09
**Physical and psychological Function**				
5 Sit-to-Stand duration (s), median (IQR)	16.70 (13.86, 20.07)	16.03 (13.61, 19.25)	17.89 (15.89, 21.49)	**.03**
TUG duration (s), median (IQR)	12.69 (10.60, 15.04)	12.31 (10.36, 14.54)	12.98 (11.56, 15.25)	.21
MoCA, median (IQR)	28 (27, 29)	28 (27, 29)	27 (24.5, 28.5)	**.01**
TMT-B (s), median (IQR)	94 (74, 120)	90 (70, 117)	100 (82, 132)	.16
HADS-A, median (IQR)	3 (1, 7)	3 (1, 5)	5 (2, 8)	**.04**
HADS-D, median (IQR)	2 (1, 4)	2 (1, 4)	3 (1, 6)	.38

Abbreviations: VOR, vestibulo-ocular reflex; MoCA, Montreal Cognitive Assessment; TMT-B, Trail Making Test-Part B; HADS-A and -D, Hospital Anxiety and Depression Scale—Anxiety and—depression; FES-I, Falls Efficacy Scale–International; TUG, Timed-Up-Go test; SD, standard deviation; IQR, interquartile range.

^a^All includes 166 participants. However, 11 participants met the criteria for concurrent perceptual–VOR elevation (see Supplementary Table 3) and were excluded from the VA group classification; therefore, the VA−/VA+ analyses include 155 participants. Bold *P*-values indicate statistically significant differences (<.05).

### Aim 1: Prevalence of vestibular agnosia

**Figure 3:**
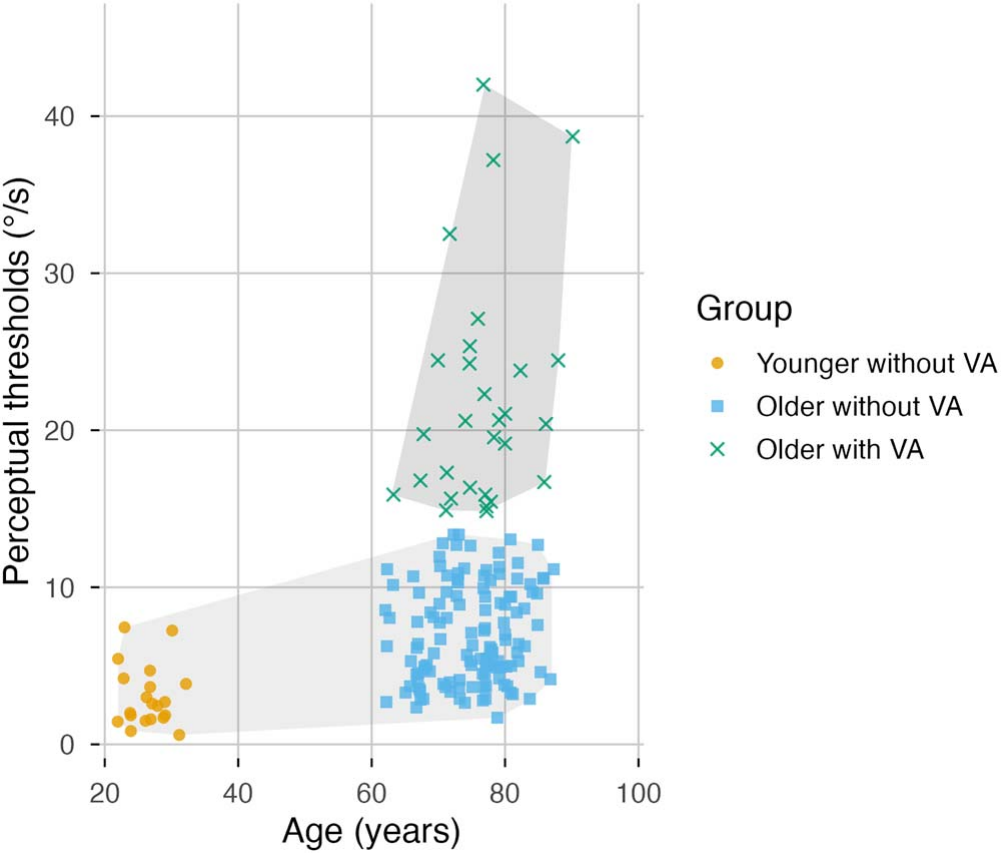
*K*-means clustering of age and average vestibular perceptual thresholds in younger (*n* = 20) and older participants (*n* = 155), classifying VA. Abbreviations: VA, vestibular agnosia.

Among the 166 older adults, 11 were classified as having concurrent perceptual–VOR elevation and were excluded from VA grouping (Supplementary Table 3). The remaining 155 with normal VOR thresholds were used to classify VA. Clustering of age and average perceptual thresholds in the full sample (young and older adults) yielded two clear clusters (silhouette coefficient: 0.69) (Figure 3): (1) a low-threshold group consisting of young adults (all 20 younger) and older adults (126/155 participants; 81.3%) and (2) a high-threshold older adult group (termed ‘VA’; 29/155 [18.7%]).

Participants with VA had—by definition—significantly higher perceptual thresholds (median: 20.4°/s vs. 6.3°/s, *P*<.001). Although we excluded participants with concurrent perceptual–VOR elevation, participants classified as VA+ nonetheless had slightly elevated VOR thresholds—albeit within ‘normal’ bounds (median: 5.4°/s vs. 3.3°/s*, P* = .006, Table 1). In unadjusted exploratory comparisons of baseline characteristics, participants with VA+ had reduced ankle vibration thresholds (*P* = .02), longer sit-to-stand duration (*P* = .03), lower cognitive function (MoCA) (*P* = .01) and higher anxiety levels (*P* = .04). Importantly, hand reaction time did not differ between groups (*P* = .89), indicating that poorer vestibular perception was not driven by reduced vigilance or slowed responding. Similarly, there were no significant differences in age, sex, executive function (TMT-B), or other demographic/health variables (Table 1).

### Aim 2: Vestibular perceptual thresholds and balance

**Figure 4:**
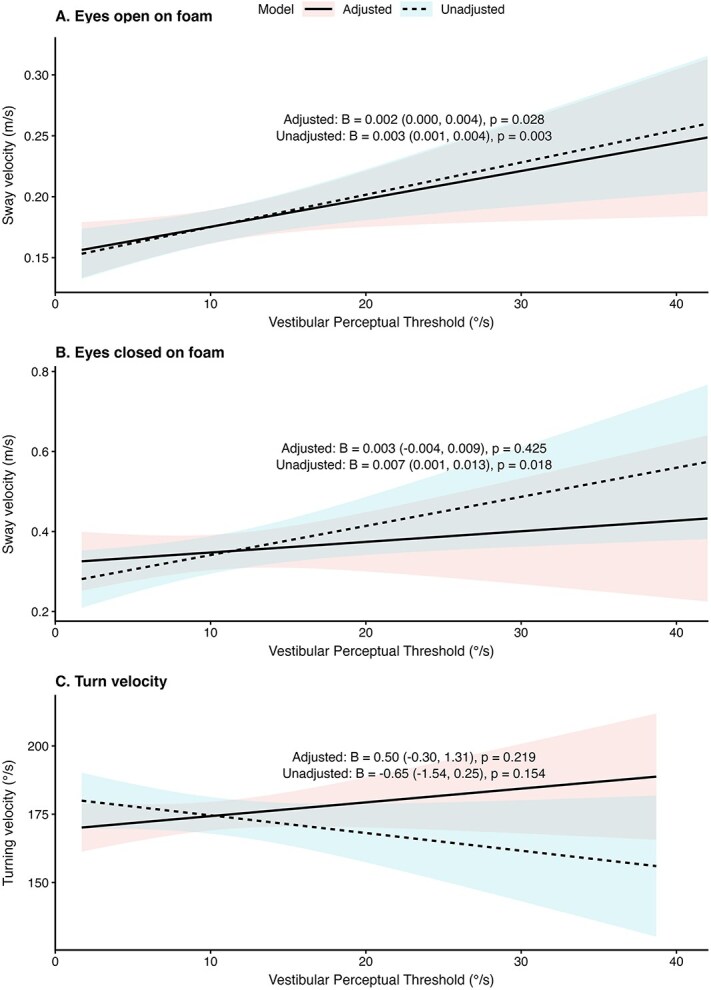
Linear regression of vestibular perceptual thresholds with sway velocity and turn velocity. Predicted values from linear regression models of vestibular perceptual threshold against (A) Sway velocity during eyes-open stance on foam; (B) Sway velocity during eyes-closed stance on foam; and (C) Turn velocity during instrumented timed up-and-go (TUG). Solid lines show models adjusted for age, sex, MoCA score, VOR thresholds, ankle vibration thresholds and 5 times sit-to-stand; dashed lines show unadjusted models. Shaded areas represent 95% confidence intervals. Note, data for turn velocity was missing for two participants.

Among the 159 participants with complete balance data, multivariable linear regression showed that higher (i.e. worse) perceptual thresholds independently related to both larger sway velocity (adjusted B [95% CI] = 0.002 [0, 0.004], *P* = .03; Figure 4A) and greater sway area when standing with eyes-open on foam (adjusted B [95% CI] = 0.002 [0, 0.003], *P* = .04; Supplementary Table 1).

Under the eyes-closed foam, higher perceptual thresholds were associated with greater sway velocity in unadjusted analyses (B [95% CI] = 0.007 [0.001, 0.013], *P* = .02), but not after adjustment (adjusted B [95% CI] = 0.003 [−0.004, 0.009], *P* = .43). Vestibular perceptual thresholds were not associated with sway area during eyes-closed foam in either the unadjusted (B [95% CI] = 0.009 [−0.022, 0.040], *P* = .58) or adjusted models (adjusted B [95% CI] = −0.019 [−0.052, 0.015], *P* = .28) (Figure 4B and Supplementary Table 1).

Perceptual thresholds were positively associated with longer TUG duration in unadjusted analysis (B [95% CI] = 0.165 [0.082, 0.249], *P* < .001), but not after adjustment (adjusted B [95% CI] = 0.028 [−0.046, 0.101], *P* = .46) (Supplementary Table 1). Perceptual thresholds were not associated with turn angle or velocity in either model (*P* > .15; Figure 4C and Supplementary Table 1).

### Aim 3: Vestibular perceptual thresholds and prospective falls

In the 161 participants (97%) who completed the full 6-month follow-up, 48 (29.8%) reported falling at least once (≥1 falls). Among those who fell, 27.1% experienced recurrent falls (≥2 falls). Faller status did not differ on either perceptual (Median: non-faller = 8.55°/s (IQR: 4.92–12.70), faller = 9.43°/s (IQR: 4.97–14.51); *P* = .66) nor VOR thresholds (Median: non-faller = 4.14°/s (IQR: 2.38–6.24), faller = 4.50°/s (IQR: 2.47–7.42); *P* = .31). We further examined the distribution of fall prevalence between the VA+ and VA– groups (11 participants with concurrent perceptual–VOR elevation were excluded). The prevalence of VA did not differ significantly between fallers (11/45; 24.4%) and non-fallers (17/105; 16.2%) (*P* = .26; Supplementary Figure 2).

Higher perceptual thresholds were not significantly associated with prospective falls (Supplementary Table 2). Perceptual thresholds were not associated with either faller status in logistic regressions (OR [95% CI] = 1.01 [0.97, 1.06], *P* = .58; adjusted OR [95% CI] = 0.99 [0.94, 1.04], *P* = .65), nor with fall counts in negative binomial models (IRR [95% CI] = 1.03 [0.99, 1.06], *P* = .11; adjusted IRR [95% CI] = 1.02 [0.97, 1.08], *P* = .35).

## Discussion

Our novel findings reveal that ~20% of community-dwelling healthy older adults in this cohort had VA. These individuals had poorer cognition, reduced lower limb somatosensation and higher anxiety. Higher (i.e. worse) vestibular perceptual thresholds were independently associated with greater imbalance when standing on foam with eyes-open. Whilst similar associations were initially observed for both eyes-closed on foam and gait performance, they were not significant after adjustment for important confounds (including VOR thresholds). Contrary to our predictions, vestibular perceptual thresholds did not predict falls over 6-month.

### Prevalence of vestibular agnosia

One-fifth of our cohort of community-dwelling older adults exhibited VA. This is consistent with a recent study that found that ~25% of older adults with BPPV reported absent dizziness [41]. VA can increase missed inner-ear vestibular diagnoses (such as BPPV) by ~7–10 times [17, 42]. Diminished symptom awareness may limit help-seeking and adaptive safety behaviours (e.g. rising slowly or limiting rapid head movements), thereby potentially increasing fall risk. Future research should explore the specific consequences of VA in older adults with peripheral vestibular disorders, including BPPV whereby the link between VA and fall risk may be more apparent.

Individuals with VA exhibited lower global cognitive performance (MoCA) in exploratory between-group comparisons. Prior research has reported similarly modest links between perceptual thresholds and specific cognitive domains (attention, fluency and language) in patients with traumatic brain injury [17]. Interestingly, VA did not relate to either executive function or vigilance in the present study, suggesting a more generalised and broad cognitive decline rather than a domain-specific deficit. This is consistent with neuroimaging evidence linking VA and related imbalance in patients with traumatic brain injury to bihemispheric fronto-posterior white matter network disruption [16].

### Vestibular perceptual thresholds and balance

Elevated (i.e. worse) perceptual thresholds were associated with increased sway velocity when standing on foam with eyes-open. These associations persisted even after adjustment for important covariates. This aligns with the prior studies linking poorer vestibular perception to increased postural sway [9–13]. Unlike prior work, our study isolates perceptual from peripheral and reflex deficits (in addition to controlling for several other relevant covariates), highlighting the independent contributions of vestibular perception to balance in healthy older adults.

Surprisingly, perceptual thresholds were not associated with balance during the most challenging condition in which vestibular contributions to balance are maximised (eyes-closed on foam), following adjustments for covariates. We focused on yaw-plane perceptual thresholds given their relevance to turning control [23] and turning-related fall risk [24], and prior evidence linking yaw-axis perception to clinically meaningful outcomes in other populations with VA (e.g. traumatic brain injury [17]). On compliant surfaces the nervous system reweights sensory input from unreliable proprioception toward visual and vestibular cues [43]. The association between yaw-plane perception and postural sway under eyes-open but not eyes-closed conditions may reflect a preferential role for yaw-plane vestibular signals when visual and vestibular cues are integrated. Under eyes-closed conditions, whilst yaw-plane cues may still contribute by detecting and correcting unintended axial rotation, overall postural stabilisation relies more on canal-otolith signals (functions partially captured by roll tilt thresholds) [10].

Although yaw-plane vestibular perceptual thresholds are mechanistically relevant to turning control [23], they were not associated with turning metrics (either turn angle or velocity). The sensory context of the task may help explain this finding. Evidence from peripheral vestibular disorders suggests that vestibular-related deficits in postural control are most apparent when alternative sensory cues are limited [44, 45]. In contrast, turning during the TUG task in the present study was performed with vision available and on a firm surface (as is standard practise). This may have allowed visual and proprioceptive cues to compensate for impaired vestibular perception. Although perceptual thresholds were associated with TUG duration in the unadjusted model, this association was attenuated after full adjustment, likely because TUG is heavily influenced by lower-limb strength and somatosensation (which were both controlled for) [46]. Future work should examine more challenging conditions, such as uneven surfaces walking or stepping over obstacles, or turning with reduced visual input.

### Vestibular perceptual thresholds and fall risk over 6-month follow-up

Contrary to our prediction, perceptual thresholds were not associated with 6-month prospective fall risk. There are several possible reasons for this. Firstly, VA is not always linked to imbalance, and can be a purely perceptual deficit [17], with neuroimaging data revealing only a partial overlap between circuits for VA and postural control [18]. Therefore, ‘VA’ may represent a mechanistically heterogeneous group, with some individuals experiencing predominantly perceptual deficits and others with combined perceptual and balance impairments. Secondly, many factors affect falls [47], including activity level and its riskiness.

Our findings differ from La Scaleia *et al*. [11], who reported that elevated pitch/roll perceptual thresholds predicted 1-year falls in older adults with type 2 diabetes. This discrepancy may reflect cohort differences, as older adults with type 2 diabetes typically have greater multisensory, metabolic [48] and brain disease burden [49] than our relatively healthy volunteers, potentially amplifying the functional impact of vestibular perceptual deficits on falls. Another important difference is that La Scaleia *et al*. [11] did not assess peripheral vestibular function. This is important because diabetes is commonly associated with peripheral vestibular dysfunction [48], making it plausible that poorer vestibular perception and increased falls are driven by peripheral vestibular dysfunction. Further, we assessed yaw-plane thresholds, while Scaleia *et al*. examined pitch/roll thresholds that may be more directly linked to postural control [7, 50]. Finally, their findings were derived from only nine fall events (9.5%), likely inflating effect estimates; whereas our higher fall rate (28.2%; 48 events) provides more stable estimates.

### Limitations

Several limitations of the present work should be considered. First, vestibular perceptual thresholds were assessed only in the yaw-plane. Although vestibular perception may reflect a stable, shared individual trait across yaw, roll and pitch axes [51, 52], age-related decline in vestibular perception may vary by axis or stimulus type [8]. Second, although we analysed all-cause falls, as is typical in falls prediction research [53, 54], we lacked structured information on the causes, contexts and mechanisms of falls. Future studies should further characterise fall circumstances, as VA may be more relevant to specific fall types or situations, such as falls during turning.

### Implications for practise

Vestibular perception testing may help characterise vestibular contributions to balance impairment [55]. Future work should test whether it adds value beyond conventional assessments for risk stratification or mechanistic phenotyping, particularly in older adults with lower cognition and greater postural sway. Given the practical challenges of routinely assessing VA with a rotating chair, preliminary evidence indicates a clinic-feasible alternative: jointly measuring perceptual and VOR responses during vestibular caloric testing [20, 56]. Future work should determine whether caloric-defined VA exhibits similar associations with behaviourally relevant outcomes as standardised rotatory chair testing.

## Conclusions

VA was identified in ~20% of healthy community-dwelling older adults, despite preserved peripheral function. Elevated vestibular perceptual thresholds were independently associated with greater sway velocity during eyes-open foam balancing (and with slower TUG in unadjusted analyses). In contrast, perceptual thresholds did not predict 6-month prospective falls, highlighting the multifactorial nature of fall risk. Overall, these findings suggest that vestibular perceptual function is independently associated with objective balance performance in older adults, but further research is needed to determine whether vestibular perceptual thresholds add predictive value for falls beyond standard balance assessments in larger, clinically representative cohorts.

## Supplementary Material

aa-26-0617-File003_afag216

## Data Availability

Individual-level data are not publicly available due to ethical and privacy restrictions. Summary data and analysis code are available from the corresponding author upon reasonable request.
